# Prognostic Role of Subclinical Congestion in Heart Failure Outpatients: Focus on Right Ventricular Dysfunction

**DOI:** 10.3390/jcm10225423

**Published:** 2021-11-20

**Authors:** Andrea Lorenzo Vecchi, Silvia Muccioli, Jacopo Marazzato, Antonella Mancinelli, Attilio Iacovoni, Roberto De Ponti

**Affiliations:** 1 Department of Medicine and Surgery, University of Insubria, 21100 Varese, Italy; j.marazzato88@gmail.com (J.M.); roberto.deponti@uninsubria.it (R.D.P.); 2 Department of Cardiology, Mauriziano Umberto I Hospital, 10128 Torino, Italy; s.muccioli89@gmail.com; 3 Department of Cardiology, ASST Papa Giovanni XXIII Hospital, 24127 Bergamo, Italy; antonella.mancinelli@yahoo.it (A.M.); aiacovoni@asst-pg23.it (A.I.)

**Keywords:** subclinical congestion, heart failure, right ventricular dysfunction, right ventricular/arterial coupling

## Abstract

Background: subclinical pulmonary and peripheral congestion is an emerging concept in heart failure, correlated with a worse prognosis. Very few studies have evaluated its prognostic impact in an outpatient setting and its relationship with right-ventricular dysfunction. The study aims to investigate subclinical congestion in chronic heart failure outpatients, exploring the close relationship between the right heart-pulmonary unit and peripheral congestion. Materials and methods: in this observational study, 104 chronic HF outpatients were enrolled. The degree of congestion and signs of elevated filling pressures of the right ventricle were evaluated by physical examination and a transthoracic ultrasound to define multiparametric right ventricular dysfunction, estimate the right atrial pressure and the pulmonary artery systolic pressure. Outcome data were obtained by scheduled visits and phone calls. Results: ultrasound signs of congestion were found in 26% of patients and, among this cohort, half of them presented as subclinical, affecting their prognosis, revealing a linear correlation between right ventricular/arterial coupling, the right-chambers size and ultrasound congestion. Right ventricular dysfunction, TAPSE/PAPS ratio, clinical and ultrasound signs of congestion have been confirmed to be useful predictors of outcome. Conclusions: subclinical congestion is widespread in the heart failure outpatient population, significantly affecting prognosis, especially when right ventricular dysfunction also occurs, suggesting a strict correlation between the heart-pulmonary unit and volume overload.

## 1. Introduction

Heart failure (HF) is a clinical syndrome with current or prior symptoms and/or signs, caused by a structural and/or functional cardiac abnormality, confirmed by elevated natriuretic peptide levels, and objective evidence of cardiogenic pulmonary or systemic congestion [1]. Congestion can be clinically overt or subclinical, and its presence in chronic HF patients is an independent predictor of outcomes [2] and disease progression [3]. An increase in left ventricular end-diastolic pressure (LVED), due to volume overload or intravascular redistribution, affects left ventricle performance, causing an increase in wall stress and, consequently, in intravascular pulmonary pressure with pulmonary congestion. An increase in the right heart afterload causes progressive tricuspid insufficiency and right ventricular dysfunction (RVD), finally resulting in the rise of right atrial pressure (RAP), progressively leading to peripheral congestion. Physical examination (PE) is the first approach used to identify congestion, but its sensitivity is extremely variable, estimated at 15–86%, while the specificity ranges from 27% to 89% [4]. Few studies focused on subclinical congestion in a chronic HF population [5,6,7], but the way in which RVD and right ventricular-arterial coupling may potentially affect congestion is still a matter of debate. The study aims to explore the effect of clinical and subclinical congestion on prognosis, focusing the analysis on its close relationship with right-ventricular function and coupling, assessed by a right focused ultrasound evaluation.

## 2. Materials and Methods

### 2.1. Study Population

We screened 110 consecutive outpatients’ data, included in the HF electronic database (Di Circolo Hospital—Macchi Foundation, Varese; Galmarini Hospital, Tradate—Portale application), from December 2018 to June 2019; only patients whose echocardiographic evaluation was performed immediately after the clinical evaluation were included in the screening. Inclusion criteria were as follows: age >18 years, previous diagnosis of HF (regardless of LVEF and etiology of heart disease); recent blood tests (previous 3 months or the following month), including creatinine, urea, hemoglobin, hematocrit, NTproBNP, sodium, potassium. Exclusion criteria were as follows: estimated glomerular filtration rate <15 mL/min/1.73 m^2^ (calculated using the Modification of Diet in Renal Disease equation); poor acoustic window; patients treated by HF telemedicine or treated with periodic or continuous inotropic infusions; recent hospitalization for HF (in the previous 30 days); acute coronary syndromes or myocardial revascularization in the previous 3 months; cardiac surgery in the previous 6 months; isolated right ventricular dysfunction and isolated tricuspid insufficiency; constrictive pericarditis; idiopathic pulmonary hypertension; severe pulmonary disease; BMI > 40; pregnancy.

A previous history of myocardial infarction or angiographic evidence of significant coronary artery disease defined the existence of ischemic heart disease. A prior medical history of hypertension defined arterial hypertension. Diabetes was considered as comorbidity in the presence of a previous diagnosis of type 1 or type 2 diabetes mellitus. Atrial fibrillation was defined as a clinical history of sustained atrial arrhythmias (atrial fibrillation, atrial flutter). Anemia was defined by a hemoglobin level of <12 g/dL in females and of <13 g/dL in males. Chronic kidney disease was defined as an estimated glomerular filtration rate of <60 mL/min/1.73 m^2^ using MDRD formula.

The study conforms to the principles outlined in the Declaration of Helsinki and was approved by the local ethics committee.

### 2.2. Physical Examination and Ultrasound Analysis

The PE was performed by two cardiologists experienced in HF. PE was systematically performed for each patient to identify clinical signs of congestion and elevated central venous pressure; the presence of these clinical signs was not graduated and was analyzed as a dichotomic classification with regard to jugular vein distention (JVD), hepato-jugular reflux (HJR), peripheral oedema (OED) and rales. JVD was systematically inspected in the supine position, at 30°–45°, on both sides of the neck, with a careful evaluation of the internal jugular venous waveform. HJR was considered as positive when a sustained increase in JVD, during 10 s of continuous pressure on the abdomen was identified, with an immediate drop after the pressure was released. A detailed clinical history, electrocardiogram and PE with vital signs were always collected before a transthoracic echocardiogram (TTE). TTE was performed in a blinded fashion by an experienced cardiologist, using a Vivid E9 (GE Healthcare, Boston, MA, US) and a Philips IE33 (Philips Healthcare, Eindhoven, NL), equipped with a cardiac probe (2.5–3.5 MHz); all measures were collected according to current guidelines. A systematic evaluation of the inferior vena cava (IVC) diameter and its collapsibility degree was performed whereby the IVC diameter was measured at end-expiration by a subxiphoid view, approximately 2 cm from venous ostium, and its collapse was estimated following deep inspiration. The RAP was estimated as 3 mmHg when an IVC diameter ≤ 21 mm and a collapsibility of > 50% were assessed; IVC diameter > 21 mm with a collapse < 50% arbitrary estimated a RAP of 13 mmHg; 8 mmHg was estimated for intermediate values of the IVC diameter and collapsibility degree. The right ventricular systolic function was assessed through a multiparametric evaluation, composed of fractional area change (FAC), tricuspid annular plane systolic excursion (TAPSE) and a systolic S’ wave, using the tissue Doppler technique. TAPSE < 17 mm, FAC < 35% and S’ < 9.5 cm/s were considered pathologic values. The tricuspid regurgitation degree has been classified by qualitative estimation as trivial, mild, moderate or severe, respectively. The systolic Pulmonary Artery Pressure (PAPS) was derived by the peak tricuspid jet velocity plus the estimated RAP; 36 mmHg was considered the upper normal range. The TAPSE/PAPS ratio was assumed as a surrogate of right ventricular–arterial coupling; values of > 0.57 were considered normal. The presence of at least two pathologic criteria among TAPSE, FAC and S’ were considered as RVD.

### 2.3. Outcome Data

The composite outcome was represented by HF rehospitalization and Emergency Department admission due to HF symptoms requiring diuretics and cardiovascular mortality.

The outcome data were obtained by scheduled visits and Emergency Department admissions using the local electronic database. All missing outcome data were obtained directly by the patients by phone call and scheduled visits. To avoid interference in the outcomes, March 2020 was considered as the end of the follow-up period, which is when the Covid-19 pandemic began.

### 2.4. Statistical Analysis 

Normally distributed continuous variables are presented as means ± standard deviation, or median and confidence intervals in case of a non-Gaussian distribution. Between-group differences were compared by a Chi square test, an Analysis of Variance test (ANOVA) and Student–Newman–Keuls, as appropriate. A Kaplan-Meier curve analysis was used to assess the event rate and for the graphic representations of outcomes. The accuracy data were expressed by area under curve (Receiver Operating Characteristic, ROC curve) and by inter-rater agreement analysis. The associations between the echographic and clinical variables were tested using multivariate logistic regression models. The correlation between variables is expressed using the Pearson correlation coefficient. A value of *p* < 0.05 was considered statistically significant.

Statistical analyses were performed using Medcalc software.

## 3. Results

### 3.1. Population

110 consecutive patients were screened from December 2018 to June 2019; 6 patients met the exclusion criteria and were removed from the final analysis.

Three groups of patients were defined:

(1) “Control” group, i.e., patients without clinical signs of peripheral edema nor US estimated RAP ≥ 13 mmHg;
(2) “SubC” (Sub Clinical Congestion) Group i.e., patients with US estimated RAP ≥ 13 mmHg without peripheral edema;
(3) “Edema” group i.e., patients with peripheral edema irrespective of US findings.

### 3.2. Patient Characteristics

Patient characteristics are summarized in Table 1.

From the analysis of the population characteristics, it was found that patients in the “Edema” group had a significantly higher mean age than the “Control” group (*p* < 0.01); no significant age difference was detected between the “Control” and “SubC” groups.

Significant differences regarding the etiology of heart disease were found: idiopathic cardiomyopathy was prevalent in the “Control” group while in the “SubC” and “Edema” groups ischemic and valvular heart disease were prevalent (*p* < 0.01).

The prevalence of both history of atrial fibrillation (AF) and permanent AF gradually increased from the “Control” group to the “Edema” group (*p* < 0.001).

No significant differences in blood chemistry were observed except for the hemoglobin parameter, which was significantly reduced in the group with clear signs of congestion (*p* < 0.01). The inter-rater agreement analysis showed a significant correlation between the circulating levels of NTproBNP and IVC diameters and collapsibility (IVC minimum diameter: correlation coefficient r 0.342, *p* < 0.01; IVC maximum diameter: correlation coefficient r 0.233, *p* < 0.05; IVC collapse: correlation coefficient r 0.338, *p* < 0.01). The baseline blood sample results are summarized in Table 2.

### 3.3. Physical Examination and Ultrasound Analysis

PE revealed pulmonary congestion in 8% of the enrolled patients, peripheral OED in 14% and signs of elevated central venous pressures (JVD and/or HJR) in 29% of the patients. Clinical or instrumental congestion was detected in 26% of the patients. In this subgroup, 12 out of 27 patients (44%) only had subclinical congestion.

RVD was found in 29% of the population and the TAPSE/PAPS ratio was reduced (i.e., <0.57) in 46% of patients. The values of TAPSE (*p* < 0.001) and FAC (*p* < 0.05) were significantly reduced in the congested groups compared to the group without instrumental and clinical congestion. Results of PE and TTE evaluations are summarized in Table 3.

**Figure 1:**
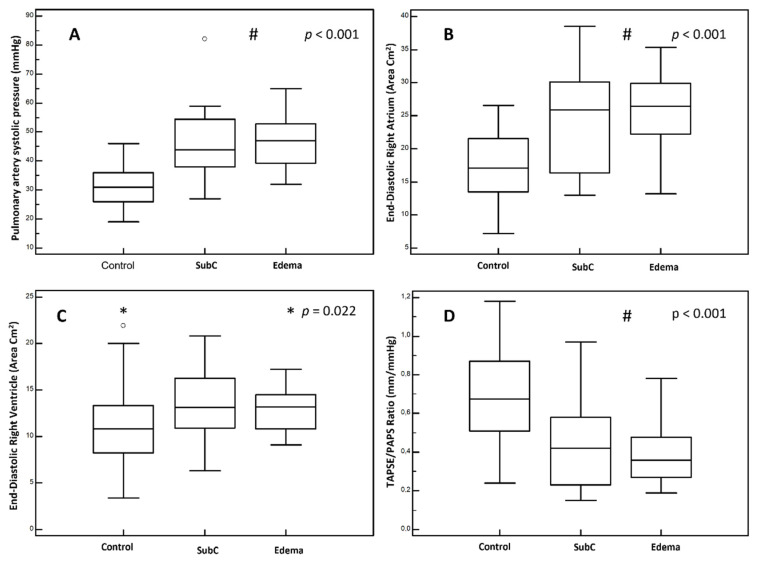
US findings, comparison between groups. (**A**): pulmonary artery systolic pressure. (**B**): end-diastolic right atrium area. (**C**): end-diastolic right ventricle area. (**D**): TAPSE/PAPS ratio. Group “Control”: patients without clinical signs of peripheral congestion nor echographic signs of congestion. Group “SubC” (Sub Clinical congestion): patients with echographic signs of congestion without peripheral clinical congestion. Group “Edema”: patients with peripheral congestion irrespective of echographic findings. #: *p* value for “SubC” and “Edema” groups vs. “Control” group. *: *p* value for selected groups.

The end-diastolic right chambers’ volume and TAPSE/PAPS showed significant difference between the congested groups compared to the group with no signs of instrumental or clinical congestion (*p* < 0.001), the results of which are summarized in Figure 1.

The PE accuracy has been analyzed by ROC curves and the combination of OED/HJR/JVD predicts US congestion with a specificity of 77% (Kappa coefficient = 0.360) and an ROC area under the curve of 0.727 (Figure S1).

The correlation analysis showed a strong correlation between both the right chamber size and ventricular/arterial coupling with US congestion. The right atrium size and TAPSE/PAPS resulted in the most significant correlations with IVC diameters and the collapse ratio.

**Figure 2:**
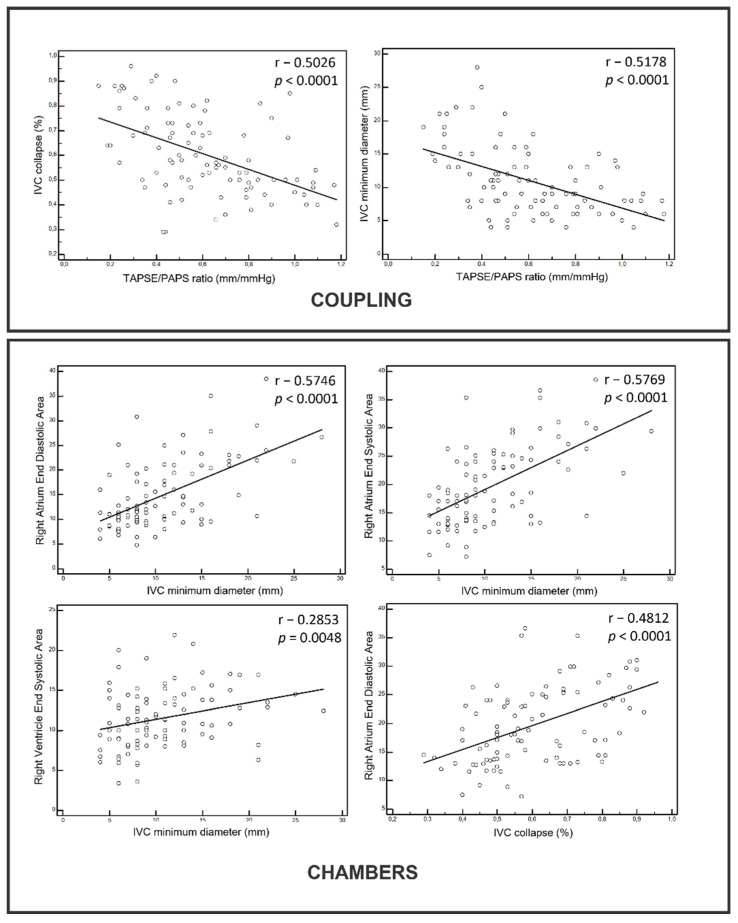
Correlations between US signs of ventricular/arterial coupling and right chambers volumes vs. congestion. IVC: Inferior Vena Cava.

The main findings are summarized in the distribution graphs below (Figure 2).

### 3.4. Outcome Data

During a follow-up of 370 days (IQR 259–448), the following 17 events occurred: 3 deaths due to cardiovascular causes and 14 Emergency Department admissions/hospitalization due to HF. Both clinical congestion, identified by PE, and instrumental congestion affect prognosis (*p* < 0.01). Pathological values of TAPSE/PAPS ratio correlated with a significant reduction of event-free survival (OR 4.5 when < 0.57 mm/mmHg; *p* = 0.01). RVD affects early prognosis, resulting in a 2-fold increase in events (OR 2.4 when at least 2 pathologic criteria among TAPSE, FAC, s’ are present; *p* = 0.05).

The combination of a multiparametric evaluation of RVD and US signs of congestion corroborates the above-mentioned findings, i.e., RVD and instrumental congestion significantly affect prognosis, which is even worse when both are present (OR 4.3, *p* < 0.05).

**Figure 3:**
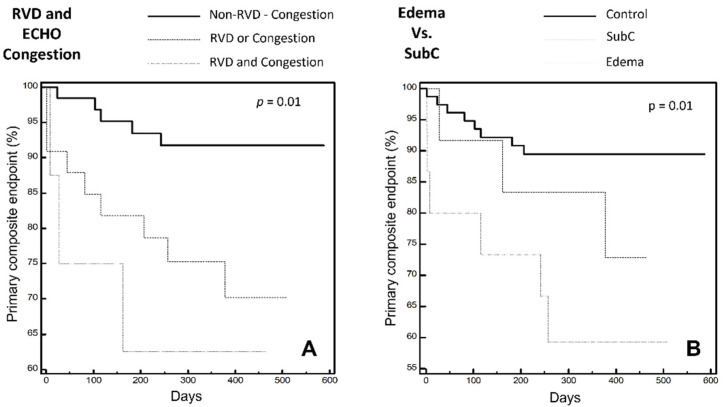
Primary composite endpoint expressed by Kaplan-Meier curves: (**A**) combining tertiles of the right ventricular dysfunction and estimated right atrial pressure. (**B**) in the three main groups: “Control”; “SubC”; “Edema”. RVD: Right Ventricular Dysfunction. SubC: Sub Clinical Congestion. ECHO Congestion: RAP ≥ 13 mmHg according to IVC diameter and collapse ratio.

Finally, by comparing the three main groups according to the presence of overt or subclinical congestion, the former is associated with the worst event rate and the latter affects prognosis even in the absence of signs of clinical congestion. According to the US and PE data analyses, the estimated survival analysis is as shown in Figure 3.

## 4. Discussion

Our study evaluated an outpatient HF population to analyze the relationship between RVD and congestion, the accuracy of PE and the prognostic impact of clinical and US variables commonly used to assess congestion in outpatient HF settings. The main population characteristics were comparable to previous HF registries [8,9] except for the presence of a slightly higher mean age and a lower percentage of HFrEF and diabetic patients, Among the HFrEF patients, 20% were prescribed ARNI, consistent with data from international registries that enrolled patients in the same period [10]. Some population differences emerged when analyzing the three groups regarding mean age, etiology, and the history of atrial fibrillation. It is noteworthy that both groups with instrumental or clinical congestion (“SubC” and “Edema”) display a higher prevalence of atrial fibrillation. This result supports the hypothesis of a more advanced disease, with a marked myocardial structural subversion; consequently, as already suggested by other authors [11], arrhythmia represents a negative and dynamic prognostic factor, especially when RVD is also present. This is consistently associated with congestion and worse outcomes, regardless of LVEF. Regarding age, patients in the edema group had a significantly higher average age, possibly associated with a worse prognosis and a longer history of disease; however, it should be noted that patients in the “SubC” group did not show significant demographic differences compared with the “Control” group, making the outcomes comparable and reinforcing the theory of an independent prognostic role of subclinical congestion, as already highlighted in several previous studies [12,13]. Furthermore, congestion is the major cause of symptoms in the patients with HF, and results in a gradually higher NYHA class in the three groups analyzed.

PE is the cornerstone of outpatient evaluation, however it often lacks accuracy compared with other instrumental evaluations [13,14], potentially leading to under/overtreatment, especially regarding diuretic strategies [6,15]. The benefits of an integrated approach combining natriuretic peptides [16] such as the easily reproducible US signs [7,12,17] and implantable device monitoring [18] are still a matter of debate [19].

Several studies described subclinical congestion in outpatient settings: B-lines [7,17], JVD ratio, IVC collapsibility and diameter [12,13] have demonstrated to be affordable and reproducible measures to predict adverse outcomes in a chronic HF population, especially when congestion signs are not overt.

The prevalence of a pathological IVC diameter and of collapsibility in our population ranges from 26% to 47% according to its grading, which is similar to other recent studies [13], highlighting the lack of accuracy of PE in clinical practice and the need for clinically affordable and relevant instrumental signs of congestion [20]. Our study confirms the low accuracy of a peripheral edema and PE in general in identifying congestion. However, half of our “SubC” patient cohort showed signs of elevated central venous pressure without showing peripheral OED. Therefore, according to recent findings [14], JVD and HJR represent clinical signs with good specificity for identifying elevated RAP; when they are detectable, they it is advisable for the clinician to investigate signs of subclinical congestion through instrumental methods. By analyzing the morpho-functional parameters of the right chambers, right atrial dilation is found to be closely associated with congestion, both clinical and instrumental. PH, RVD and ventricular/arterial decoupling represent the triggering and maintenance factors of the right chamber’s dilation and should be considered as an indirect US sign of elevated pulmonary pressure and RAP, measured invasively, as already noted in previous studies [11,21]. This dynamic condition is associated with the further worsening of the right ventricular function, regardless of LVEF, triggering a vicious circle of “chamber dilation-increased filling pressures-tricuspid insufficiency-arrhythmias”. In this context, when comparing non-congested patients with the “SubC” and “Edema” cohort, we note that the right ventricular function parameters in the three groups show a significant difference. It is even more important to note that the parameters of ventricular/arterial coupling and of RVD are substantially identical in the congested groups, with overlapping values in the “SubC” and “Edema” groups. These data support the thesis that when subclinical congestion is present, it is associated with RVD and ventricular/arterial decoupling, even in the absence of over fluid clinical signs. The correlation analysis between the US signs of congestion and the morpho-functional characteristics of the “right heart-pulmonary unit” suggest a close relationship between fluid overload and ventricular/arterial decoupling, showing an almost linear relationship between cavity dimensions, the TAPSE/PAPS ratio and the IVC diameters.

Data from the literature identifies RVD as a strong predictor of outcomes in the HF population, irrespective of LVEF [22,23]. Ghio et al. observed that the prognosis of RVD patients without PH was considerably better than for patients with RVD and PH, assuming that PH and ventricular/arterial coupling act as the main determinant of the prognosis [22]. In this study, the TAPSE/PAPS ratio is confirmed to be a strong predictor of outcome due to its comprehensive evaluation of ventricular/arterial coupling rather than the right ventricular function alone. When instrumental signs of overt congestion are detectable, the prognosis is dramatically affected. By combining data from the right ventricular function and instrumental congestion, when RVD or elevated estimated RAP are detected, we can identify a subset of poor prognosis patients irrespective of PE. When both RVD and elevated estimated RAP are present, long-term prognosis significantly drops after one year follow-up, similarly to the lower TAPSE/PAPS tertiles groups, according to previous studies [24].

Finally, in agreement with previous observations, the presence of subclinical congestion allows us to identify a group of patients at high risk of events, even in the absence of clinical signs of congestion; this group is identifiable only by instrumental examination, presenting very similar ultrasound characteristics to patients with clinically overt signs of congestion. Due to the poor prognosis of the “SubC” cohort, it is mandatory to implement subclinical congestion detection methods in the HF outpatient population.

## 5. Conclusions

Despite the fact that congestion represents an important therapeutic target and prognostic factor in HF outpatients, PE has a low sensitivity for identifying congestion, resulting in a significant proportion of subclinical congested patients. Subclinical congestion is a negative prognostic factor, strongly correlated with the right heart structure and ventricular/arterial coupling, amplifying RVD negative outcomes. When congestion coexists with RVD, it dramatically impacts prognosis, even if it is subclinical. An integrated, focused US approach provides accurate prognostic information and could allow physicians to empower the clinical patient risk assessment, potentially guiding future therapeutic approaches, according to patients’ characteristics. Further analyses are necessary to evaluate whether tailored therapy in subclinical congested patients could have a favorable impact on prognosis in an outpatient setting.

## 6. Limits

The main limitations of the study are its small sample size, the lack of external validation of the data acquired in the US laboratory, the lack of a control group without HF and the absence of the invasive validation of US measurements. Furthermore, the presence of pulmonary congestion has not been systematically evaluated by US examination.

The sample size was mainly limited by the requirement to enroll patients who had an adequate observation period with a deadline of February 2020, when the Covid-19 pandemic started, as this would have interfered with the primary endpoints. The sample size calculation obtained before starting enrollment estimated the range of patients to be enrolled between 81 and 120; therefore, the 104 enrollments made it possible to obtain a statistically significant result for almost all the data analyzed. The analysis of the literature has shown that the correlation between US, clinical and hemodynamic data is already well established; for this reason it was not considered necessary to acquire the data of control patients nor to carry out invasive measurements. Moreover, clinical congestion revealed by the PE has not been graded as it has been in recent studies, although it has been identified dichotomously, which could affect the accuracy and the odds ratio analyzes. However, this study aimed to analyze the correlation between instrumental congestion and RVD, both graded in tertiles according to previous studies, which has not been influenced by PE grading. Finally, systematic data on tricuspid regurgitation severity and liver function were insufficient and were therefore omitted from the analysis.

## Figures and Tables

**Table 1:** Main clinical patients’ characteristics divided into subgroups under comparison. Group “Control”: patients without clinical signs of peripheral congestion nor echographic signs of congestion. Group “SubC” (Sub Clinical congestion): patients with echographic signs of congestion without peripheral clinical congestion. Group “Edema”: patients with peripheral congestion irrespective of echographic findings.

Population Characteristics	Total Pop (*n* 104)	Control (*n* 77)	SubC (*n* 12)	Edema (*n* 15)	*p*-Value
Sex (male)	72 (69%)	54 (70%)	9 (75%)	9 (60%)	ns
Age (years)	73 ± 11	70 ± 11	75 ± 11	81 ± 4	<0.01 #
Weight (Kg)	76 ± 15	76 ± 15	77 ± 21	74 ± 14	ns
Height (cm)	170 ± 9	170 ± 9	171 ± 6	170 ± 11	ns
BMI (Kg/m^2^)	26 ± 4	26 ± 4	26.4 ±7.0	25.5 ±3.3	ns
BSA (m^2^)	1.86 ± 0.20	1.86 ± 0.21	1.89 ±0.21	1.85 ±0.22	ns
NYHA class					<0.001
I	29 (28%)	25 (32%)	3 (25%)	1 (7%)	
II	54 (52%)	44 (57%)	5 (42%)	5 (33%)	
III	20 (19%)	8 (10%)	4 (33%)	8 (53%)	
IV	1 (1%)	0 (0%)	0 (0%)	1 (7%)	
LVEF < 40%	44 (42%)	34 (44%)	5 (42%)	5 (33%)	ns
Etiology					<0.01
Ischemic	35 (34%)	24 (31%)	5 (42%)	6 (40%)	
Valvular	13 (12%)	6 (8%)	2 (17%)	5 (33%)	
Idiopathic	38 (37%)	36 (47%)	2 (17%)	0 (0%)	
Miscellaneous	18 (17%)	11 (14%)	3 (25%)	4 (27%)	
Months since HF diagnosis	45 (16–106)	49 (19–107)	16 (7–81)	19 (15–61)	ns
Comorbidities					
Stroke in past	5 (5%)	4 (5%)	1 (8%)	0 (0%)	ns
Previous cardiac surgery	15 (14%)	13 (17%)	0 (0%)	2 (13%)	ns
Previous mitral valve repair or clip	9 (8%)	6 (8%)	2 (17%)	1 (7%)	ns
Diabetes	26 (25%)	19 (25%)	3 (25%)	4 (27%)	ns
History of cancer	16 (15%)	10 (13%)	3 (25%)	3 (20%)	ns
Hypertension	71 (68%)	49 (64%)	10 (83%)	12 (80%)	ns
Peripheral artery disease	5 (5%)	4 (5%)	0 (0%)	1 (7%)	ns
History of atrial fibrillation	43 (42%)	24 (31%)	7 (58%)	12 (80%)	<0.001
Permanent atrial fibrillation	25 (24%)	12 (16%)	5 (42%)	8 (53%)	<0.001
Therapy					
ACEi	68 (65%)	50 (65%)	7 (58%)	11 (73%)	ns
ARB	14 (13%)	10 (13%)	3 (25%)	1 (7%)	ns
BetaB	96 (91%)	72 (94%)	12 (100%)	12 (80%)	ns
Ivabradine	12 (11%)	11 (14%)	1 (8%)	0 (0%)	ns
Digoxin	5 (5%)	3 (4%)	1 (8%)	1 (7%)	ns
MRA	48 (46%)	34 (44%)	7 (58%)	7 (47%)	ns
NTG	7 (7%)	5 (6%)	2 (17%)	0 (0%)	ns
Warfarin	34 (33%)	18 (23%)	6 (50%)	10 (67%)	<0.001
NOACs	9 (8%)	4 (5%)	2 (17%)	3 (20%)	<0.05
Statins	53 (50%)	42 (55%)	7 (58%)	4 (27%)	ns
Furosemide	83 (80%)	59 (77%)	10 (83%)	14 (93%)	ns
Amiodarone	24 (23%)	17 (22%)	2 (17%)	5 (33%)	ns
ARNI	9 (8%)	7 (9%)	1 (8%)	1 (7%)	ns
ICD	34 (32%)	30 39%)	2 (17%)	2 (13%)	ns
CRT	9 (8%)	8 (10%)	1 (8%)	0 (0%)	ns

(#) = (1) vs. (3) Student-Newman-Keuls test was used for all pairwise comparisons *p* < 0.05.

**Table 2:** Blood sample results divided into subgroups under comparison. Group “Control”: patients without clinical signs of peripheral congestion nor echographic signs of congestion. Group “SubC” (Sub Clinical congestion): patients with echographic signs of congestion without peripheral clinical congestion. Group “Edema”: patients with peripheral congestion irrespective of echographic findings.

Population Characteristics	Total Pop (*n* 104)	Control (*n* 77)	SubC (*n* 12)	Edema (*n* 15)	*p*-Value
Blood tests					
Hematocrit (%)	41.3 (39.1–43.9)	41.2 (39.1–44.0)	41.2 (39.0–43.8)	41.0 (38.9–43.5)	ns
Hemoglobin (g/dL)	13.7 (12.7–14.6)	13.7 (12.7–14.6)	13.2 ± 1.7	11.9 (11.3–13.5)	<0.01
Creatinine (mg/dL)	1.2 (1.0–1.5)	1.2 (1.0–1.5)	1.2 (1.1 -1.3)	1.4 (1.1–1.6)	ns
eGFR MDRD (mL/min/1.73 m^2^)	57 (46–73)	58 (47–77)	56 (48–65)	55 (46–66)	ns
NTproBNP pg/mL	944 (237–1755)	716 (192–1548)	1361 (764–2172)	2116 (1111–4945)	ns
Sodium (mEq/L)	141 (139–143)	141 (140–143)	142 (141–143)	141 (138–144)	ns
Potassium (mEq/L)	4.5 (4.2–4.9)	4.5 (4.2–4.8)	4.5 (4.4–4.8)	4.5 (4.3–4.9)	ns

**Table 3:** Main PE and echographic characteristics divided into subgroups under comparison. Group “Control”: patients without clinical signs of peripheral congestion nor echographic signs of congestion. Group “SubC” (Sub Clinical congestion): patients with echographic signs of congestion without peripheral clinical congestion. Group “Edema”: patients with peripheral congestion irrespective of echographic findings.

Population Characteristics	Total Pop (*n* 104)	Control (*n* 77)	SubC (*n* 12)	Edema (*n* 15)	*p*-Value
Vital signs					
Heart rate (bpm)	69 ± 13	70 ± 12	69 ± 10	69 ± 16	ns
Systolic blood pressure	127 ± 17	129 ± 16	123 ± 13	125 ± 21	ns
Diastolic blood pressure	75 ± 10	76 ± 10	72 ± 7	74 ± 12	ns
Physical examination					
Pulmonary congestion	8 (8%)	1 (1%)	2 (17%)	5 (33%)	<0.001
Peripheral congestion	15 (14%)	0 (0%)	0 (0%)	15 (100%)	<0.001
Elevated CVP	30 (29%)	11 (14%)	6 (50%)	13 (87%)	<0.001
Ultrasound parameters					
LVEF (%)	40 ± 11	40 ± 11	40 ± 12	42 ± 13	
TAPSE (mm)	20 ± 5	21 ± 5	18 ± 7	18 ± 6	<0.05
PAPS (mmHg)	35 ± 11	26 ± 5	47 ± 14	47 ± 11	<0.001 *
TAPSE/PAPS	0.63 ± 0.26	0.71 ± 0.23	0.43 ± 0.24	0.40 ± 0.16	<0.001 *
S’VD (cm/sec)	10.6 ± 2.8	11.0 ± 2.7	8.9 ± 3.2	10.0 ± 3.1	= 0.05
RV end-diastolic area (cm^2^)	19.1 ± 4.6	18.7 ± 5.0	20.1 ± 3.8	20.0 ± 2.6	ns
RV end-systolic area (cm^2^)	11.5 ± 3.7	10.9 ± 3.7	13.4 ± 4.0	13.0 ± 2.5	< 0.05 #
RV FAC	0.40 ± 0.13	45 ± 13	33 ± 16	35 ± 11	<0.05
RA end-diastolic area (cm^2^)	14.8 ± 6.7	12.4 ± 4.5	20.5 ± 8.9	21.5 ± 7.0	0.001 *
RA end-systolic area (cm^2^)	19.6 ± 6.8	17.4 ± 5.1	24.8 ± 8.5	25.7 ± 6.9	0.001 *
IVC Min (mm)	11 ± 5	8 ± 3	19 ± 4	22 ± 5	<0.001 °
IVC Max (mm)	17 ± 5	15 ± 4	24 ± 3	16 ± 4	<0.001 *
IVC collapse (%)	40 ± 16	45 ± 13	22 ± 12	25 ± 11	<0.001 *

(#) = (1) vs. (3) Student-Newman-Keuls test for all pairwise comparisons *p* < 0.05. (*) = (1) vs. both (2) and (3) Student-Newman-Keuls test for all pairwise comparisons *p* < 0.05. (°) = (1) vs. (2) vs. (3) Student-Newman-Keuls test for all pairwise comparisons *p* < 0.05.

## Data Availability

The data presented in this study are available on request from the corresponding author.

## Supplementary Materials

The following are available online at https://www.mdpi.com/article/10.3390/jcm10225423/s1: Figure S1: accuracy of PE expressed by ROC curves.

## References

[1] Bozkurt B., Coats A.J., Tsutsui H., Abdelhamid C.M., Adamopoulos S., Albert N., Anker S.D., Atherton J., Böhm M., Butler J. (2021). Universal definition and classification of heart failure: A report of the Heart Failure Society of America, Heart Failure Association of the European Society of Cardiology, Japanese Heart Failure Society and Writing Committee of the Universal Definition of Heart Failure: Endorsed by the Canadian Heart Failure Society, Heart Failure Association of India, Cardiac Society of Australia and New Zealand, and Chinese Heart Failure Association. Eur. J. Heart Fail..

[2] Lucas C., Johnson W., Hamilton M.A., Fonarow G., Woo M.A., Flavell C.M., Creaser J.A., Stevenson L.W. (2000). Freedom from congestion predicts good survival despite previous class IV symptoms of heart failure. Am. Heart J..

[3] Pellicori P., Cleland J.G.F., Zhang J., Kallvikbacka-Bennett A., Urbinati A., Shah P., Kazmi S., Clark A.L. (2016). Cardiac Dysfunction, Congestion and Loop Diuretics: Their Relationship to Prognosis in Heart Failure. Cardiovasc. Drugs Ther..

[4] Thibodeau J., Drazner M.H. (2018). The Role of the Clinical Examination in Patients with Heart Failure. JACC Heart Fail..

[5] Curbelo J., Aguilera M., Rodriguez-Cortes P., Gil-Martinez P., Fernández C.S. (2018). Usefulness of inferior vena cava ultrasonography in outpatients with chronic heart failure. Clin. Cardiol..

[6] Öhman J., Harjola V.-P., Karjalainen P., Lassus J. (2018). Assessment of early treatment response by rapid cardiothoracic ultrasound in acute heart failure: Cardiac filling pressures, pulmonary congestion and mortality. Eur. Heart J.-Acute Cardiovasc. Care.

[7] Platz E., Merz A., Jhund P., Vazir A., Campbell R., Mcmurray J. (2017). Dynamic changes and prognostic value of pulmonary congestion by lung ultrasound in acute and chronic heart failure: A systematic review. Eur. J. Heart Fail..

[8] Maggioni A.P. (2015). Epidemiology of Heart Failure in Europe. Heart Fail. Clin..

[9] Chioncel O., Lainscak M., Seferovic P.M., Anker S.D., Crespo-Leiro M.G., Harjola V.-P., Parissis J., Laroche C., Piepoli M.F., Fonseca C. (2017). Epidemiology and one-year outcomes in patients with chronic heart failure and preserved, mid-range and reduced ejection fraction: An analysis of the ESC Heart Failure Long-Term Registry. Eur. J. Heart Fail..

[10] Vaduganathan M., Fonarow G.C., Greene S.J., DeVore A.D., Kavati A., Sikirica S., Albert N.M., Duffy C.I., Hill C.L., Patterson J.H. (2020). Contemporary Treatment Patterns and Clinical Outcomes of Comorbid Diabetes Mellitus and HFrEF: The CHAMP-HF Registry. JACC Heart Fail..

[11] Obokata M., Reddy Y.N.V., Melenovsky V., Pislaru S., Borlaug B.A. (2019). Deterioration in right ventricular structure and function over time in patients with heart failure and preserved ejection fraction. Eur. Heart J..

[12] Pellicori P., Carubelli V., Zhang J., Castiello T., Sherwi N., Clark A.L., Cleland J.G. (2013). IVC diameter in patients with chronic heart failure: Relationships and prognostic significance. JACC Cardiovasc. Imaging.

[13] Pellicori P., Shah P., Cuthbert J., Urbinati A., Zhang J., Kallvikbacka-Bennett A., Clark A.L., Cleland J.G. (2019). Prevalence, pattern and clinical relevance of ultrasound indices of congestion in outpatients with heart failure. Eur. J. Heart Fail..

[14] Pham D.D., Drazner M.H., Ayers C.R., Grodin J.L., Hardin E.A., Garg S., Mammen P.P.A., Amin A., Araj F.G., Morlend R.M. (2021). Identifying Discordance of Right- and Left-Ventricular Filling Pressures in Patients With Heart Failure by the Clinical Examination. Circ. Heart Fail..

[15] Yavaşi Ö., Ünlüer E.E., Kayayurt K., Ekinci S., Sağlam C., Sürüm N., Köseoğlu M.H., Yeşil M. (2014). Monitoring the response to treatment of acute heart failure patients by ultrasonographic inferior vena cava collapsibility index. Am. J. Emerg. Med..

[16] Mueller C., McDonald K., de Boer R.A., Maisel A., Cleland J.G., Kozhuharov N., Coats A.J., Metra M., Mebazaa A., Ruschitzka F. (2019). Heart Failure Association of the European Society of Cardiology practical guidance on the use of natriuretic peptide concentrations. Eur. J. Heart Fail..

[17] Curbelo J., Rodriguez-Cortes P., Aguilera M., Gil-Martinez P., Martín D., Fernandez C.S. (2019). Comparison between inferior vena cava ultrasound, lung ultrasound, bioelectric impedance analysis, and natriuretic peptides in chronic heart failure. Curr. Med Res. Opin..

[18] Lindenfeld J., Zile M.R., Desai A.S., Bhatt K., Ducharme A., Horstmanshof D., Krim S.R., Maisel A., Mehra M.R., Paul S. (2021). Haemodynamic-guided management of heart failure (GUIDE-HF): A randomised controlled trial. Lancet.

[19] Khan A., Khan D., Shadi M., MacDougall K., Lafferty J. (2020). Utilization of Ultrasound to Assess Volume Status in Heart Failure. J. Clin. Med. Res..

[20] Pellicori P., Platz E., Dauw J., ter Maaten J.M., Martens P., Pivetta E., Cleland J.G., McMurray J.J., Mullens W., Solomon S.D. (2021). Ultrasound imaging of congestion in heart failure: Examinations beyond the heart. Eur. J. Heart Fail..

[21] Schmeißer A., Rauwolf T., Groscheck T., Fischbach K., Kropf S., Luani B., Tanev I., Hansen M., Meißler S., Schäfer K. (2021). Predictors and prognosis of right ventricular function in pulmonary hypertension due to heart failure with reduced ejection fraction. ESC Heart Fail..

[22] Ghio S., Guazzi M., Scardovi A.B., Klersy C., Clemenza F., Carluccio E., Temporelli P.L., Rossi A., Faggiano P., Traversi E. (2017). Different correlates but similar prognostic implications for right ventricular dysfunction in heart failure patients with reduced or preserved ejection fraction. Eur. J. Heart Fail..

[23] Gorter T.M., Hoendermis E.S., Van Veldhuisen D.J., Voors A.A., Lam C.S.P., Geelhoed B., Willems T.P., Van Melle J.P. (2016). Right ventricular dysfunction in heart failure with preserved ejection fraction: A systematic review and meta-analysis. Eur. J. Heart Fail..

[24] Gorter T.M., Van Veldhuisen D.J., Voors A.A., Hummel Y.M., Lam C.S., Berger R.M., Van Melle J.P., Hoendermis E.S. (2017). Right ventricular-vascular coupling in heart failure with preserved ejection fraction and pre- vs. post-capillary pulmonary hypertension. Eur. Heart J.-Cardiovasc. Imaging.

