# Supplementary figures and images for: Prognostic Role of Subclinical Congestion in Heart Failure Outpatients: Focus on Right Ventricular Dysfunction
Source: J Clin Med. 2021 Nov 20;10(22):5423. doi: 10.3390/jcm10225423 (PMC8625381; doi:10.3390/jcm10225423)

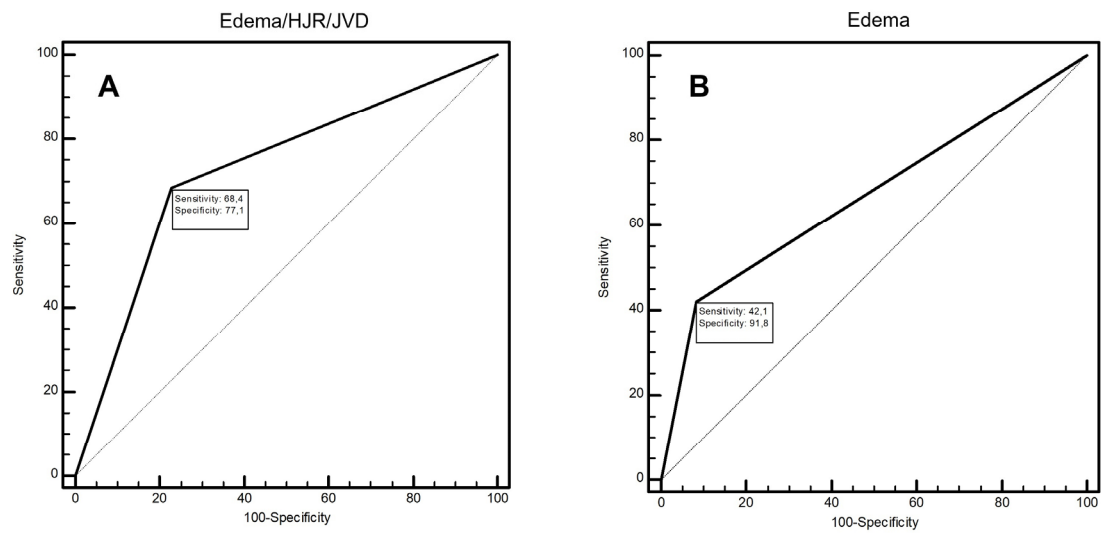

**Figure S1.** accuracy of PE expressed by ROC curves.

Supplement: Supplementary file 1 [file jcm-10-05423-s001.zip › jcm-1428606-supplementary.pdf]
